# Deep learning-based diffusion tensor cardiac magnetic resonance reconstruction: a comparison study

**DOI:** 10.1038/s41598-024-55880-2

**Published:** 2024-03-07

**Authors:** Jiahao Huang, Pedro F. Ferreira, Lichao Wang, Yinzhe Wu, Angelica I. Aviles-Rivero, Carola-Bibiane Schönlieb, Andrew D. Scott, Zohya Khalique, Maria Dwornik, Ramyah Rajakulasingam, Ranil De Silva, Dudley J. Pennell, Sonia Nielles-Vallespin, Guang Yang

**Affiliations:** 1 National Heart and Lung Institute, Imperial College London, London, SW7 2AZ UK; 2 Cardiovascular Research Centre, Royal Brompton Hospital, London, SW7 2AZ UK; 3 Bioengineering Department and Imperial-X, Imperial College London, London, W12 7SL UK; 4 Department of Computing, Imperial College London, London, UK; 5 Department of Applied Mathematics and Theoretical Physics, University of Cambridge, Cambridge, UK

**Keywords:** Deep learning, CNN, Transformer, Cardiac diffusion tensor, MRI reconstruction, Biomedical engineering, Cardiovascular diseases

## Abstract

In vivo cardiac diffusion tensor imaging (cDTI) is a promising Magnetic Resonance Imaging (MRI) technique for evaluating the microstructure of myocardial tissue in living hearts, providing insights into cardiac function and enabling the development of innovative therapeutic strategies. However, the integration of cDTI into routine clinical practice poses challenging due to the technical obstacles involved in the acquisition, such as low signal-to-noise ratio and prolonged scanning times. In this study, we investigated and implemented three different types of deep learning-based MRI reconstruction models for cDTI reconstruction. We evaluated the performance of these models based on the reconstruction quality assessment, the diffusion tensor parameter assessment as well as the computational cost assessment. Our results indicate that the models discussed in this study can be applied for clinical use at an acceleration factor (AF) of $\times 2$ and $\times 4$, with the D5C5 model showing superior fidelity for reconstruction and the SwinMR model providing higher perceptual scores. There is no statistical difference from the reference for all diffusion tensor parameters at AF $\times 2$ or most DT parameters at AF $\times 4$, and the quality of most diffusion tensor parameter maps is visually acceptable. SwinMR is recommended as the optimal approach for reconstruction at AF $\times 2$ and AF $\times 4$. However, we believe that the models discussed in this study are not yet ready for clinical use at a higher AF. At AF $\times 8$, the performance of all models discussed remains limited, with only half of the diffusion tensor parameters being recovered to a level with no statistical difference from the reference. Some diffusion tensor parameter maps even provide wrong and misleading information.

## Introduction

In vivo cardiac diffusion tensor (DT) imaging (cDTI) is an emerging Magnetic Resonance Imaging (MRI) technique that has the potential to describe the microstructure of myocardial tissue in living hearts. The diffusion of water molecules occurs anisotropically due to the microstructure of the myocardium, which can be approximated by fitting three-dimensional (3D) tensors with a specific shape and orientation in cDTI. Various parameters can be derived from the DT, including mean diffusivity (MD) and fractional anisotropy (FA), which are crucial indices that can indicate the structural integrity of myocardial tissues. The helix angle (HA) signifies local cell orientations, while the second eigenvector (E2A) represents the average sheetlet orientation^1^. The development of cDTI provides insights into the myocardial microstructure and offers new perspectives on the elusive connection between cellular contraction and macroscopic cardiac function^1,2^. Furthermore, it presents opportunities for novel assessments of the myocardial microstructure and cardiac function, as well as the development and evaluation of innovative therapeutic strategies^3^. Early exploratory clinical studies, e.g., cardiomyopathy^1,2,4^, myocardial infarction^5,6^, have been reported, and shown very promising results and high potential for cDTI to contribute to the clinic.

Despite the numerous advantages, there are still significant technical obstacles that must be overcome to integrate cDTI into routine clinical practice. For the calculation of the DT, diffusion-weighted images (DWIs) with diffusion encoding in at least six distinct directions need to be collected. Due to the movement derived from heart beats and human breath, in vivo cDTI exploits single-shot encoding acquisition for repetitive fast scanning, e.g., single-shot echo planar imaging (SS-EPI) or spiral diffusion-weighted imaging^7^. The utilisation of these single-shot encoding acquisitions, leading to low signal-to-noise ratio (SNR) images, typically requires multiple repetitions to enhance the accuracy of the DT estimation^8,9^. Each repetition necessitates an additional breath-hold for the patient when using breath-hold acquisitions, which significantly increases the total scanning time and leads to uncomfortable patient experience.

Numerous studies have been proposed to accelerate cDTI technique, which can be mainly categorised as (1) reducing the total amount of DWIs used for the calculation of the DT; (2) general fast DWI by *k*-space undersampling and reconstruction using compressed sensing (CS) or deep learning techniques. It is noted that this study focuses on the second strategy.

Deep learning has emerged as a powerful technique for image analysis, capitalising on the non-linear and complex nature of networks through supervised or unsupervised learning, and has found widespread applications in medical image research^10^. Deep learning-based MRI reconstruction^11,12^ has gained significant attention, leveraging its capability of learning complex and hierarchical representations from large MRI datasets^13^.

In this work, we investigated the application of deep learning-based methods for cDTI reconstruction. We explored and implemented three representative deep learning-based models from algorithm unrolling models^14–16^, enhancement-based models^17–20^ to emerging generative models^21–23^, on the cDTI dataset with acceleration factor (AF) of $\times 2$, $\times 4$ and $\times 8$, including a Convolutional Neural Network (CNN)-based algorithm unrolling method, i.e., D5C5^15^, a CNN-based and conditional Generative Adversarial Network (GAN)-based method, i.e., DAGAN^21^, and a Transformer-based and enhancement-based method, i.e., SwinMR^19^. The performance of these models was evaluated by the reconstruction quality assessment and the DT parameters assessment.

To the best of our knowledge, this work is the first comparison study that focuses on evaluating various deep learning-based models on in vivo cDTI, encompassing algorithm unrolling models, enhancement-based models, and generative models. The purpose of this work is not to propose a new reconstruction model for cDTI. Instead, it aims to validate the performance of existing MRI reconstruction models on the DWI reconstruction and to provide a general framework for a fair comparison.

Our experiments demonstrate that the models discussed in this paper can be applied for clinical use at AF $\times 2$ and AF $\times 4$, since both the reconstruction of DWIs and DT parameters reach satisfactory levels. Among these models, D5C5 shows superior fidelity for the reconstruction, while SwinMR provides results with higher perceptual scores. There is no statistical difference from the reference for all the DT parameters at AF $\times 2$ or most of the DT parameters at AF $\times 4$. The quality of most the DT parameter maps we considered are visually acceptable. Considering various factors, SwinMR is recommended as the optimal approach for the reconstruction with AF $\times 2$ and AF $\times 4$.

However at AF $\times 8$, the performance of these three models, including the best-performing SwinMR, is still limited. The reconstruction quality is unsatisfactory due to the artefact remaining and the noisy (DAGAN) or hallucinated (SwinMR) estimation. Only half of the DT parameters can be recovered to a level that is no statistical difference from the reference. Some DT parameter maps even provide wrong and misleading information, which is unacceptable and dangerous for clinical use.

## Related works

### Diffusion tensor MRI acceleration

A major drawback of DTI is its extended scanning time, as it requires multiple DWIs with varying *b*-value and diffusion gradient directions to calculate the DT. In theory, the estimation of the DT requires only six DWIs with different diffusion gradient directions and one reference image. However, in practical for cDTI, a considerable number of cardiac DWIs and multiple averages are typically required to enhance the accuracy of DT estimation, due to the inherently low SNR of single-shot acquisitions.

Strategies to accelerate the DTI technique have been explored. One technical route aims to reduce the number of DWIs required for the DT estimation^24–30^, which can be further categorised into three sub-class.

1. Learn a direct mapping from DWIs with reduced number of repetitions (or gradient directions), to the DT or DT parameter maps. Ferreira et al.^24^ proposed a U-Net^31^-based model for cDTI acceleration, which directly estimated the DT, using DWIs collected within one breath-hold, instead of solving a conventional linear-least-square (LLS) tensor fitting. Karimi et al.^25^ introduced a Transformer-based model with coarse-and-fine strategy to provide accuracy estimation of the DT, using only six diffusion-weighted measurements. Aliotta et al.^32^ proposed a neural network for brain DTI, namely DiffNet, which estimated MD and FA maps directly from diffusion-weighted acquisitions with as few as three diffusion-encoding directions. They further improved their method by combining a parallel U-Net for slice-to-slice mapping and a multi-layer perceptron for pixel-to-pixel mapping^26^. Li et al.^27^ developed a CNN-based model for brain DTI, i.e., SuperDTI, to generate FA, MD and directionally encoded color maps with as few as six diffusion-weighted acquisitions.
2. Enhance DWIs (denoising). This kind of methods usually apply only a small amount of enhanced images to achieve comparable estimation results with the results reconstructed using a standard protocol. Tian et al.^28^ developed a novel DTI processing framework, entitled DeepDTI, which minimised the required data for DTI to six diffusion-weighted images. The core idea of this framework was to use a CNN that took a stack of non-diffusion-weighted (*b0*) image, six DWIs as well as a anatomical (T1- or T2-weighted) image as input, to produce high-quality *b0* images and six DWIs. Phipps et al.^29^ applied a denoising CNN to enhance the quality of *b0* images and corresponding DWIs for cDTI.
3. Refine the DT quality. Tänzer et al.^30^ proposed a GAN-based Transformer, aiming to directly enhance the quality of the DT that was calculated with a reduce number of DWIs in an end-to-end manner.

Another technical route follows the general DWIs acceleration by *k*-space undersampling and reconstruction^33–36^. Zhu et al.^33^ directly estimated the DT from highly undersampled *k*-space data. Chen et al.^34^ incorporated a joint sparsity prior of different DWIs with the *L*1-*L*2 norm and the DT’s smoothness with the total variational (TV) semi-norm to efficiently expedite DWI reconstruction. Huang et al.^35^ utilised a local low-rank model and 3D TV constraints to reconstruct the DWIs from undersampled *k*-space measurements. Teh et al.^36^ introduced a directed TV-based method for DWI images reconstruction, applying the information on the position and orientation of edges in the reference image.

In addition to these major technical routes, Liu et al.^37^ explored deep learning-based image synthetics for inter-directional DWIs generation. The true *b0* and 6 DWIs were concatenated with the generated data and passed to the CNN-based tensor fitting network.

### Deep learning-based reconstruction

The aim of MRI reconstruction is to recover the image of interest ${\textbf{x}} \in {\mathbb {C}}^n$ from the undersampled *k*-space measurement ${\textbf{y}} \in {\mathbb {C}}^m$, which is mathematically described as an inverse problem:

1 $$\begin{aligned} \begin{aligned} {\hat{\textbf{x}}} = {\text {arg}}\min _{{\textbf{x}}} \frac{1}{2}|| {\textbf{A}} {\textbf{x}} - {\textbf{y}} ||_2^2 + \lambda {\mathcal {R}}({\textbf{x}}), \end{aligned} \end{aligned}$$

in which the degradation matrix ${\textbf{A}} \in {\mathbb {C}}^{m \times n}$ can be further presented as the combination of the undersampling trajectory ${\textbf{M}} \in {\mathbb {C}}^{m \times n}$, discrete Fourier transform matrix $\boldsymbol{\mathcal{F}}\in {\mathbb {C}}^{n \times n}$ and a diagonal matrix that denotes coil sensitivity maps ${\textbf{S}} \in {\mathbb {C}}^{n \times n}$. $\lambda$ is the coefficient that balances regularisation term ${\mathcal {R}}({\textbf{x}})$.

Deep learning technique has been widely used for MRI reconstruction, among which the three most representative methods are (1) algorithm unrolling models^14–16^, (2) enhancement-based models^17–20^, and the emerging (3) generative models^21–23.^

Algorithm unrolling models typically integrate neural networks with traditional CS algorithms, simulating the iterative reconstruction algorithms through learnable iterative blocks^12^. Yang et al.^14^ reformulated an Alternating Direction Method of Multipliers (ADMM) algorithm to a multi-stage deep architecture, namely Deep-ADMM-Net, for MRI reconstruction, of which each stage corresponds to an iteration in traditional ADMM algorithm. Some algorithm unrolling-based models defined the regulariser with the denoising residual of a deep denoiser^15,16^, where Eq. (1) can be reformulated as:

2 $$\begin{aligned} \begin{aligned} {\hat{\textbf{x}}} = {\text {arg}}\min _{{\textbf{x}}} \frac{1}{2}|| {\textbf{A}} {\textbf{x}} - {\textbf{y}} ||_2^2 + \lambda || {\textbf{x}} - {\textbf{f}}_{\theta } ({\textbf{x}}_u) ||_2^2, \quad \text {s. t. } {\textbf{x}}_u = {\textbf{A}}^{H} {\textbf{y}}, \end{aligned} \end{aligned}$$

in which ${\textbf{f}}_{\theta }(\cdot )$ is a deep neural network and ${\textbf{x}}_u$ is the undersampled zero-filled images (ZF). Schlemper et al.^15^ designed a deep cascade of CNNs for cardiac cine reconstruction, in which a spatio-temporal correlations can be also efficiently learned via the data sharing approach. Aggarwal et al.^16^ proposed a model-based deep learning method, namely MoDL, which exploited a CNN-based regularisation prior with a conjugate gradient-based data consistency (DC) for MRI reconstruction.

Enhancement-based models typically train a deep neural network ${\textbf{f}}_{\theta }(\cdot )$ that directly maps the undersampled *k*-space measurement ${\textbf{y}}$ or zero-filled images ${\textbf{x}}_u$, to fully-sampled images ${\hat{\textbf{x}}}$ or its residual in an end-to-end manner, which can be formulated as ${\hat{\textbf{x}}} = {\textbf{f}}_{\theta }({\textbf{x}}_u)$ or ${\hat{\textbf{x}}} = {\textbf{f}}_{\theta }({\textbf{x}}_u) + {\textbf{x}}_u$. Hyun et al.^17^ introduced a CNN-based U-Net for MRI reconstruction. Feng et al.^18^ exploited the task-specific novel cross-attention and designed an end-to-end Transformer-based model for jointly MRI reconstruction and super-resolution. Huang et al.^20^ explored the graph representation and the non-Euclidean relationship for MR images, and designed a Vision Graph Neural Network-based U-Net for fast MRI.

Generative models for solving inverse problems represent an emerging and rapidly evolving field of data-driven models. In the area of MRI reconstruction, various models and techniques have been found and achieved promising performance^21–23,38^. Generative models usually focus on learning the true data distribution, instead of heavily replying upon the regularisation term or directly learning an inverse mapping^39^. For example, Variational Autoencoders^40^ explicitly learn the data distribution by utilising variational inference to approximate the posterior distribution of latent variables, given the observed data. Generative Adversarial Networks (GANs)^41^ are able to learn the data distribution implicitly by minimising a statistical discrepancy between the true distribution and the model distribution in a max-min game. Diffusion models^42^ approximate the gradient of the log probability density of the data, focusing on the score function rather than the density itself^39^. In the context of MRI reconstruction, Yang et al.^21^ proposed a de-aliasing Generative Adversarial Networks for MRI reconstruction, in which the U-Net-based generator produced the estimated fully-sampled MRI images in an end-to-end manner. Chung  et al.^22^ introduced a score-based diffusion model for MRI reconstruction with an arbitrary undersampling rate, taking advantage of unconditional training strategy and data consistency conditioning at the inference stage.

Deep learning community constantly provides a wide range of novel and powerful network structures for these MRI reconstruction methods, including CNNs^15,17,21,43^, Recurrent Neural Networks^44,45^, Graph Neural Networks^20^, recently thriving Transformers^18,19,46,47^, etc. These rapidly evolving deep learning-based networks enable advances in MRI reconstruction.

## Methodology

**Figure 1:**
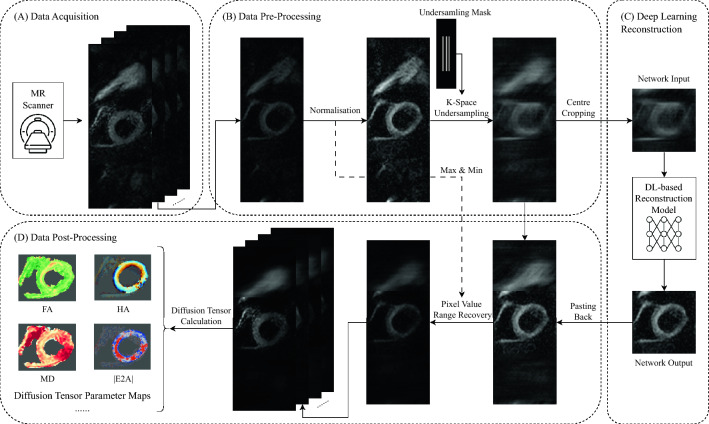
The data flow of our implementation for cardiac diffusion tensor imaging data. The whole procedure consists (**A**) data acquisition, (**B**) data pre-processing, (**C**) deep learning-based reconstruction, and (**D**) data post-processing. It is noted that D5C5 does not require the cropping and pasting step and additionally takes the undersampled *k*-space data and the corresponding undersampling mask as input.

In this study, we implemented three deep learning-based MRI reconstruction methods, namely, DAGAN^21^, D5C5^15^ and SwinMR^19^, and assessed their performance on a cDTI dataset. The overall data flow is depicted in Fig. 1.

### Data acquisition

All data used in this study were approved by the National Research Ethics committee, Bloomsbury with reference number 13/LO/1830, REC reference 10/H0701/112, and IRAS reference number 33773. The study adheres to the principles of the Declaration of Helsinki and the UK Research Governance Framework version 2. All participants provided informed written consent.

Retrospectively acquired cDTI data were acquired using Siemens Skyra 3T MRI scanner and Siemens Vida 3T MRI scanner (Siemens AG, Erlangen, Germany). A diffusion-weighted stimulated echo acquisition mode (STEAM) SS-EPI sequence with reduced phase field-of-view and fat saturation was used. Some MR sequence parameters are listed: $\text {TR} = 2 \ \text {RR intervals}$; $\text {TE} = 23 \ \text {ms}$; mSENSE or GRAPPA with $\text {AF} = 2$; echo train duration $= 13 \ \text {ms}$; spatial resolution $= 2.8 \times 2.8 \times 8.0 \ \text {mm}^\text {3}$. Diffusion-weighted images were encoded in six directions with diffusion-weighted of $\text {b} = 150 \ \text {and} \ 600 \ \text {sec/mm}^\text {2}$ (namely b150 and b600) in a short-axis mid-ventricular slice. Reference images, namely *b0*, were also acquired with a minor diffusion weighting.

We used 481 cDTI cases including 2 cardiac phases, i.e., diastole ($n = 232$) and systole ($n = 249$), for the experiments section. The dataset contains 241 healthy cases, 31 amyloidosis (AMYLOID) cases, 47 dilated cardiomyopathy (DCM) cases, 35 in-recovery DCM (rDCM) cases, 39 hypertrophic cardiomyopathy (HCM) cases, 48 HCM genotype-positive-phenotype-negative (HCM G+P-) cases, and 40 acute myocardial infarction (MI) cases. The overall data distribution of our dataset is shown in Table 1. The detailed data distribution per cohort and cardiac phase can be found in Supplementary (Supp.) Table S4.

This work separately discussed the reconstruction of systole and diastole cases. For each deep learning-based methods, two network weights were trained for either systole or diastole reconstruction. In the training stage, we applied a 5-fold-cross-validation strategy, using 169 diastole cases (TrainVal-D) or 183 systole cases (TrainVal-S). In the testing stage, four testing sets were utilised, including a mixed ordinary testing set with diastole cases (Test-D) or systole cases (Test-S) and an out-of-distribution MI testing set with diastole cases (Test-MI-D) or systole cases (Test-MI-S). According to Supp. Table S4, Test-D and Test-S include the data of Health, AMYLOID, rDCM, DCM, HCM and HCM G+P-, which are also included in the TrainVal. For further examining the model robustness and ability to handle out-of-the-distribution data, Test-MI dataset includes only MI cases, which were ‘invisible’ for models during the training stage.

**Table 1:** The overview of the dataset.

Cardiac phase	Total		TrainVal		Test		Test-MI	
	Case	Slice	Case	Slice	Case	Slice	Case	Slice
Diastole	272	22509	169	14182	43	3630	20	1470
Systole	290	23654	183	15054	46	3938	20	1470

### Data pre-processing

In the data pre-processing stage, all DWIs (*b0*, b150 and b600) were processed following the same protocol.

The pixel intensity ranges of DWIs vary considerably across different *b*-values. To address this, We normalised all DWIs in the dataset to a pixel intensity range of $0 \sim 1$ using the max-min method, while the maximum and minimum pixel values of all DWIs were recorded for the pixel intensity range recovery at the beginning of the data post-processing stage.

In our dataset, the majority of DWIs have a resolution of $256 \times 96$, while a small subset of 2D slices exhibit a resolution of $256 \times 88$. *red* In order to standardise the resolution, zero-padding was conducted, turning the images with a resolution of $256 \times 88$ to a resolution of $256 \times 96$.

GRAPPA-like Cartesian *k*-space undersampling masks with AF $\times 2$, $\times 4$ and $\times 8$, generated by the official protocol of fastMRI dataset^13^, were applied to simulate the *k*-space undersampling process. Since all the 2D slices have been reconstructed with zero-padding factor of two, the phase encoding (PE) of our undersampling masks was set to 48 instead of 96, for a more realistic simulation. The undersampling masks were then zero-padded from $128 \times 48$ to $256 \times 96$ as shown in Fig. 1. More details regarding the undersampling masks can be found in Supp. Figure S1.

For DAGAN and SwinMR, DWIs were further cropped to $96 \times 96$, as both models only support square-shaped input images.

### Deep learning-based cardiac diffusion tensor imaging reconstruction

We implemented and evaluated three deep learning-based models, encompassing an algorithm unrolling model, namely D5C5^15^, an enhancement-based model, namely SwinMR^19^, and a generative model (for solving inverse problem), namely DAGAN^21^.

#### DAGAN

**Figure 2:**
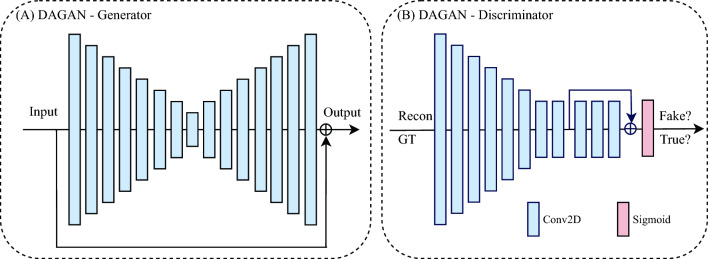
The model architecture of DAGAN. (**A**) the generator of DAGAN is a modified Convolutional Neural Network (CNN)-based U-Net with a residual connection; (**B**) the discriminator of DAGAN is a standard CNN-based classifier. Conv2D: 2D convolutional layer; Recon: reconstructed MR images; GT: ground truth MR images.

DAGAN^21^ is a conditional GAN-based and CNN-based model designed for general MRI reconstruction, of which the model structure is presented in Fig. 2. DAGAN comprises two components: a generator and a discriminator, which are trained in an adversarial manner as a two-player game. The generator is a modified CNN-based U-Net^31^ with a residual connection^48^, which takes the ZF MR image as input and aims to produce the reconstructed MR image as close as possible to the ground truth image (GT). The discriminator is a standard CNN-based classifier that attempts to distinguish the ‘fake’ reconstructed MR images generated by the generator, from the ground truth MR images.

DAGAN can be trained with a hybrid loss function including an image space *L*2 loss, a frequency space *L*2 loss, a perceptual *L*2 loss based on a pre-trained VGG^49^, as well as an adversarial loss^41^ The generator and discriminator were alternatively updated in the training stage, while in the inference stage, only the generator was applied. To adapt DAGAN to the cDTI dataset, except for changing the resolution, we reduced the convolution kernel size of the generator and the discriminator, to lighten the network size, while ensuring the performance. Further implementation details can be found in Supp. Table S1.

#### D5C5

**Figure 3:**
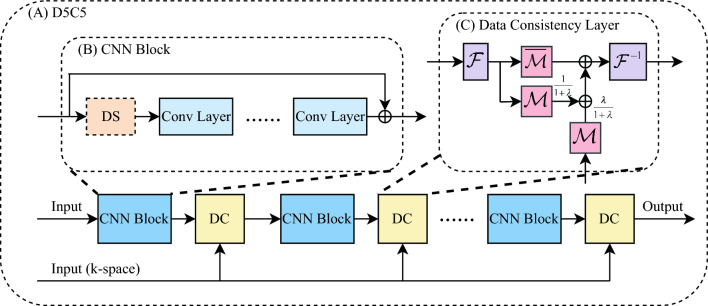
(**A**) The model architecture of D5C5. D5C5 has five stages, each comprising a Convolutional Neural Network block (CNN Block) and a data consistency layer (DC). (**B**) The structure of the CNN Block. One optional data sharing module (DS) and five convolutional layers (Conv Layers) are included in the CNN Block. (**C**) The structure of the DC. ${\mathcal {M}}$ denotes the undersampling mask, and $\overline{{\mathcal {M}}} = {\mathcal {I}} - {\mathcal {M}}$. ${\mathcal {F}}$ and ${\mathcal {F}}^{-1}$ denote the Fourier and inverse Fourier transform. $\lambda$ is an adjustable coefficient controlling the level of DC. It is noted that DS module is not applied for our task.

D5C5^15^ is a CNN-based model for MRI reconstruction, with its model structure presented in Fig. 3. D5C5 takes the undersampled *k*-space measurement as well as the ZF MR image as the input, and outputs the reconstructed MR image. It is composed of multiple stages, each comprising a CNN block and a DC layer. The CNN block contains a cascade of convolutional layers with Rectified Linear Units (ReLU) for feature extraction, an optional data sharing (DS) layer for learning spatio-temporal features, as well as a residual connection^48^. The DC layer takes a linear combination between the output of the CNN block and the undersampled *k*-space data, enforcing the consistency between the prediction of CNNs and the original *k*-space measurement.

Although D5C5 is built in an iterative style, similar to other unrolling models, it can be trained in an end-to-end manner, using an image space *L*2 loss function. To adapt D5C5 for our experiment, we removed the DS module in each stage, since D5C5 was originally proposed for cine MRI reconstruction. Further implementation details can be found in Supp. Table S2.

#### SwinMR

**Figure 4:**
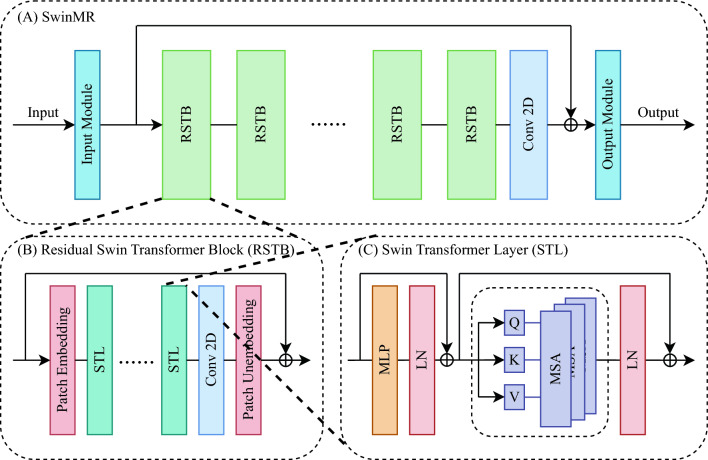
(**A**) The model architecture of SwinMR. (**B**) The structure of the residual Swin Transformer block (RSTB). (**C**) The structure of the Swin Transformer layer (STL). Conv2D: 2D convolutional layer. MLP: multi-layer perceptron. LN: layer normalisation. Q: query; K: key; V: value. MSA: multi-head self-attention.

SwinMR^19^ is a Swin Transformer-based model for MRI reconstruction, with its model structure shown in Fig. 4. SwinMR takes the ZF MR image as the input and directly outputs the reconstructed image. SwinMR is composed of a CNN-based input module and output module for projecting between the image space and the latent space, a cascade of residual Swin Transformer blocks (RSTBs), and a convolution layer with a residual connection for feature extraction. A patch embedding and a patch unembedding layer are placed at the beginning and end of each RSTB, facilitating the inter-conversion of feature maps and sequences, since the computation of Transformers is based on sequences. Multiple standard Swin Transformer layers (STLs)^50^ and a single convolutional layer are applied between the patch embedding and unembedding layer.

SwinMR can be trained end-to-end with a hybrid loss function consisting of an image space Charbonnier loss, a frequency space Charbonnier loss, a perceptual *L*1 loss based on a pre-trained VGG^49^. Further implementation details can be found in Supp. Table S3.

### Data post-processing

We applied our in-house developed software (MATLAB 2021b, MathWorks, Natick, MA) for cDTI post-processing, following the protocol described in^1,24^. The post-process procedure for reference data includes: (1) manual removal of low-quality DWIs; (2) DWI registration; (3) semi-manual segmentation for left ventricle (LV) myocardium; (4) DT calculation via the LLS fit; (5) DT parameter calculation including FA, MD, HA and E2A. The initial post-processing of reference data was performed by either Z.K. (7 years of experience), R.R. (3 years of experience) or M.D. (2 years of experience), and subsequently reviewed by P.F. (10 years of experience).

For the post-processing of deep learning-based reconstruction results, the output ($96 \times 96$) of DAGAN and SwinMR were ‘pasted’ back to the corresponding zero-filled images ($256 \times 96$) at their original position. (This process does not affect the final post-processing results since the region of interest is set in the central $96 \times 96$ area).

All the DWIs were ‘anti-normalised’ (pixel value range recovery) to their original pixel intensity range using the maximum and minimum values recorded in the pre-processing stage.

The reconstruction results were arranged to construct a new reconstruction dataset with the same structure as the reference dataset. The reconstructed dataset was then automatically post-processed following the configuration of the reference data (e.g., low-quality removal information, registration shifting, segmentation masks) for a fair comparison.

## Experiments and results

In this section, the experimental results are presented from three perspectives: (1) the quality of DWI reconstruction, (2) the quality of DT parameter estimation and (3) the assessment of computational cost.

### Reconstruction quality assessment

In this study, four metrics were considered to assess the reconstruction quality. Peak Signal-to-Noise Ratio (PSNR) is a simple and commonly used metric for measuring the reconstruction quality, which measures the ratio of the maximum possible power of a signal to the power of corrupting noise. A higher PSNR value indicates a better reconstruction quality. Structural Similarity Index (SSIM) is a perceptual-based metric that measures the similarity between two images by comparing their structural information. A higher SSIM value indicates a better reconstruction quality. Learned Perceptual Image Patch Similarity (LPIPS)^51^ is a learned metric that measures the perceptual similarity between two images by computing the distance in the latent space using a pre-trained deep neural network. LPIPS has shown a high correlation with human perceptual judgements of the image similarity. A lower LPIPS value indicates a better generated images quality. Fréchet Inception Distance (FID)^52^ is a learned metric that measures the similarity between two sets of images by comparing their feature statistics, using a pre-trained deep neural network. FID has also shown to have high correlation with human perceptual experience. A lower FID value indicates a better generated images quality.

**Table 2:** The quantitative reconstruction results on the testing sets Test-S and Test-D with undersampling masks of the acceleration factor (AF) $\times 2$, $\times 4$ and $\times 8$. SSIM, PSNR and LPIPS results are quoted as ‘mean (standard deviation)’. ^⋆^ indicates the specific distribution is significantly different ($p < 0.05$) from the **best-resulting** distribution by the two-sample t-test.

Metrics	Test-S				Test-D			
AF $\times$2	ZF	DAGAN	D5C5	SwinMR	ZF	DAGAN	D5C5	SwinMR
SSIM $\uparrow$	0.819 (0.042) ^⋆^	0.857 (0.026) ^⋆^	**0.931 (0.030)**	0.919 (0.031) ^⋆^	0.819 (0.044) ^⋆^	0.851 (0.025) ^⋆^	**0.932 (0.031)**	0.921 (0.034) ^⋆^
PSNR $\uparrow$	25.76 (2.27) ^⋆^	27.86 (1.59) ^⋆^	**31.80 (2.45)**	30.83 (2.50) ^⋆^	26.41 (2.38) ^⋆^	28.25 (1.52) ^⋆^	**32.43 (2.70)**	31.61 (2.80) ^⋆^
LPIPS $\downarrow$	0.149 (0.037) ^⋆^	0.060 (0.024) ^⋆^	**0.050 (0.026)**	**0.050 (0.023)**	0.148 (0.034) ^⋆^	0.066 (0.028) ^⋆^	0.053 (0.032)	**0.052 (0.029)**
FID $\downarrow$	82.29	33.1	18.53	**17.2**	101.55	38.84	25.83	**22.7**

**AF** $\times$**4**	ZF	DAGAN	D5C5	SwinMR	ZF	DAGAN	D5C5	SwinMR
SSIM $\uparrow$	0.663 (0.068) ^⋆^	0.751 (0.039) ^⋆^	**0.849 (0.044)**	0.842 (0.048) ^⋆^	0.668 (0.065) ^⋆^	0.783 (0.039) ^⋆^	**0.860 (0.047)**	0.851 (0.051) ^⋆^
PSNR $\uparrow$	20.92 (2.34) ^⋆^	24.20 (1.62) ^⋆^	**26.85 (2.14)**	26.56 (2.19) ^⋆^	21.86 (2.40) ^⋆^	25.66 (1.76) ^⋆^	**28.27 (2.37)**	27.75 (2.51) ^⋆^
LPIPS $\downarrow$	0.321 (0.040) ^⋆^	0.117 (0.041) ^⋆^	0.092 (0.030) ^⋆^	**0.090 (0.030)**	0.313 (0.041) ^⋆^	0.090 (0.032)	0.091 (0.039) ^⋆^	**0.089 (0.039)**
FID $\downarrow$	218.18	61.9	38.42	**29.48**	212.61	51.41	44.64	**36.17**

**AF** $\times$**8**	ZF	DAGAN	D5C5	SwinMR	ZF	DAGAN	D5C5	SwinMR
SSIM $\uparrow$	0.529 (0.084) ^⋆^	0.579 (0.054) ^⋆^	0.680 (0.067) ^⋆^	**0.719 (0.074)**	0.544 (0.080) ^⋆^	0.595 (0.054) ^⋆^	0.689 (0.064) ^⋆^	**0.720 (0.072)**
PSNR $\uparrow$	18.26 (2.37) ^⋆^	20.15 (1.73) ^⋆^	21.52 (2.09) ^⋆^	**22.32 (2.19)**	19.33 (2.40) ^⋆^	20.99 (1.80) ^⋆^	22.63 (2.20) ^⋆^	**23.17 (2.30)**
LPIPS $\downarrow$	0.491 (0.040) ^⋆^	0.212 (0.059) ^⋆^	0.197 (0.049) ^⋆^	**0.165 (0.046)**	0.473 (0.037) ^⋆^	0.199 (0.061) ^⋆^	0.196 (0.055) ^⋆^	**0.166 (0.055)**
FID $\downarrow$	375.91	100.14	127.37	**62.28**	368.58	85.79	137.83	**72.96**

**Figure 5:**
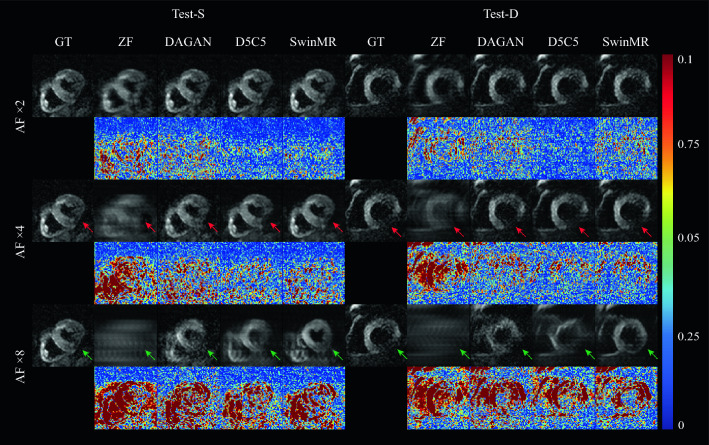
The visualised samples of the reconstruction on Test-S and Test-D with the undersampling masks of AF $\times 2$, $\times 4$ and $\times 8$. Odd Rows: the ground truth (GT), undersampled *k*-space zero-filled images (ZF) and the reconstruction results of DAGAN, D5C5 and SwinMR. Even Rows: the corresponding difference ($\times 10$) of ZF and the reconstruction results of DAGAN, D5C5 and SwinMR. Row 1–2: AF $\times 2$; Row 3–4: AF $\times 4$; Row 5–6: AF $\times 8$; Col 1–5: the results on testing set Test-S; Col 6–10: the results on testing set Test-D.

Quantitative reconstruction results on the Test-S and Test-D datasets are presented in Table 2. A two-sample t-test was applied for the statistical analysis, and ^⋆^ in Table 2 indicates that the specific result distribution is significantly different ($p < 0.05$) from the **best-resulting** distribution. Among the evaluated models, D5C5 demonstrates superior fidelity in the reconstruction, while SwinMR provides results with higher perceptual score.

Visualised samples of the reconstruction results on Test-S and Test-D datasets are shown in Fig. 5.

### Diffusion tensor parameter quality assessment

We further evaluated the quality of DT parameters, including FA, MD, E2A and HA, after post-processing.

**Table 3:** Differences of diffusion tensor parameter global mean values between the reference and reconstruction results (undersampled *k*-space zero-filled images ZF included), on systole testing sets Test-S and Test-MI-S. Mean absolute error are applied for fractional anisotropy (FA), mean diffusivity (MD), and mean absolute angular error are applied for helix angle gradient (HA Slope) and second eigenvector (E2A). The results are quoted as ‘median [interquartile range]’. ^⋆^ indicates the specific error distribution is significantly different from the **best-resulting** distribution by Mann–Whitney Test ($p < 0.05$). Data point with a green background indicates that the specific distribution of corresponding diffusion tensor parameter global mean values is NOT significantly different from the reference distribution by Mann–Whitney Test ($p > 0.05$). Units: FA unitless; MD $10^{-3} \, \text {mm}^{2}\, \text {sec}^{-1}$; HA Slope $\text {degrees} \, \text {mm}^{-1}$ and E2A degrees.

DT Para.	Test-S				Test-MI-S			
AF $\times 2$	ZF	DAGAN	D5C5	SwinMR	ZF	DAGAN	D5C5	SwinMR
FA	0.057 [0.021] ^⋆^	0.006 [0.008]	**0.005 [0.008]**	0.008 [0.007]	0.075 [0.016] ^⋆^	**0.011 [0.014]**	0.012 [0.009]	0.012 [0.007]
MD	0.011 [0.012] ^⋆^	0.008 [0.007] ^⋆^	**0.003 [0.006]**	**0.003 [0.009]**	0.027 [0.014] ^⋆^	0.005 [0.008]	**0.002 [0.006]**	0.004 [0.004]
HA Slope	1.264 [0.849] ^⋆^	0.244 [0.279] ^⋆^	0.227 [0.228]	**0.165 [0.244]**	1.560 [0.630] ^⋆^	0.220 [0.503]	0.208 [0.166]	**0.164 [0.161]**
E2A	2.658 [4.213] ^⋆^	0.850 [1.248] ^⋆^	**0.570 [0.858]**	0.608 [1.197]	3.093 [4.065] ^⋆^	0.904 [1.284]	0.959 [0.993]	**0.699 [0.805]**

**AF** $\times 4$	ZF	DAGAN	D5C5	SwinMR	ZF	DAGAN	D5C5	SwinMR
FA	0.140 [0.043] ^⋆^	**0.014 [0.020]**	0.031 [0.028] ^⋆^	0.020 [0.019]	0.167 [0.043] ^⋆^	**0.029 [0.022]**	0.056 [0.020] ^⋆^	0.033 [0.029]
MD	0.034 [0.028] ^⋆^	0.022 [0.041] ^⋆^	0.013 [0.020]	**0.011 [0.014]**	0.076 [0.041] ^⋆^	0.026 [0.028] ^⋆^	**0.009 [0.017]**	0.013 [0.019] ^⋆^
HA Slope	2.608 [1.080] ^⋆^	0.492 [0.569]	0.747 [0.728] ^⋆^	**0.274 [0.433]**	2.952 [1.084] ^⋆^	0.575 [0.546]	0.905 [0.820] ^⋆^	**0.229 [0.368]**
E2A	10.365 [9.306] ^⋆^	1.977 [2.747]	**1.661 [2.874]**	1.680 [1.557]	7.372 [6.349] ^⋆^	2.393 [2.551]	3.444 [3.890] ^⋆^	**2.372 [1.878]**

**AF** $\times 8$	ZF	DAGAN	D5C5	SwinMR	ZF	DAGAN	D5C5	SwinMR
FA	0.210 [0.049] ^⋆^	**0.034 [0.036]**	0.048 [0.041] ^⋆^	0.045 [0.050] ^⋆^	0.240 [0.037] ^⋆^	**0.051 [0.038]**	0.122 [0.043] ^⋆^	0.072 [0.053]
MD	0.049 [0.045] ^⋆^	0.052 [0.054] ^⋆^	0.039 [0.051] ^⋆^	**0.031 [0.028]**	0.139 [0.069] ^⋆^	0.066 [0.071] ^⋆^	**0.028 [0.035]**	0.039 [0.057]
HA Slope	3.839 [1.937] ^⋆^	2.073 [1.495] ^⋆^	2.537 [2.229] ^⋆^	**1.273 [1.611]**	4.131 [1.124] ^⋆^	2.358 [1.666]	2.767 [1.496]	**2.327 [1.973]**
E2A	14.346 [9.715] ^⋆^	6.359 [8.273] ^⋆^	4.847 [7.203]	**2.942 [5.299]**	11.849 [8.061] ^⋆^	4.916 [9.433]	5.000 [6.944]	**4.334 [7.459]**

Differences in DT parameter global mean values between the reference and reconstruction, on systole testing sets (Test-S and Test-MI-S) and diastole testing sets (Test-D and Test-MI-D) are presented in Table 3 and Supp. Table S6, respectively. The mean absolute error for FA, MD and the mean absolute angular error for the HA gradient (HA Slope) and E2A were employed to quantify the difference. The Mann–Whitney test was utilised for the statistical analysis, and ^⋆^ in Table 3 and Supp. Table S6 indicates that the specific error distribution is significantly different ($p < 0.05$) from the **best-resulting** distribution. Data point with a green background indicates that the specific distribution of corresponding DT parameter global mean values is NOT significantly different ($p > 0.05$) from the reference distribution according to the Mann–Whitney Test.

Overall, SwinMR achieved better or comparable (not significantly different) MD, HA slope and E2A results on all testing sets. DAGAN achieved better or comparable (not significantly different) FA results on all testing sets. D5C5 provided better results only on Test-S at AF $\times 2$, but it is not significantly better than SwinMR (on MD, HA Slope and E2A) and DAGAN (on FA).

**Figure 6:**
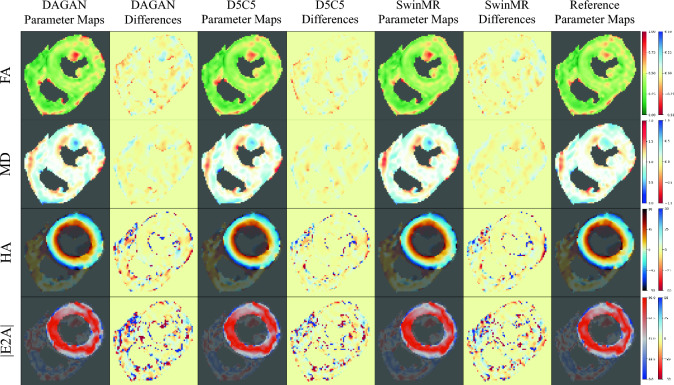
Diffusion parameter maps of the reconstruction results (AF $\times 2$) and the reference of a healthy systole case from testing set Test-S. Row 1: fractional anisotropy (FA); Row 2: mean diffusivity (MD); Row 3: helix angle (HA); Row 4: absolute value of the second eigenvector (|E2A|).

**Figure 7:**
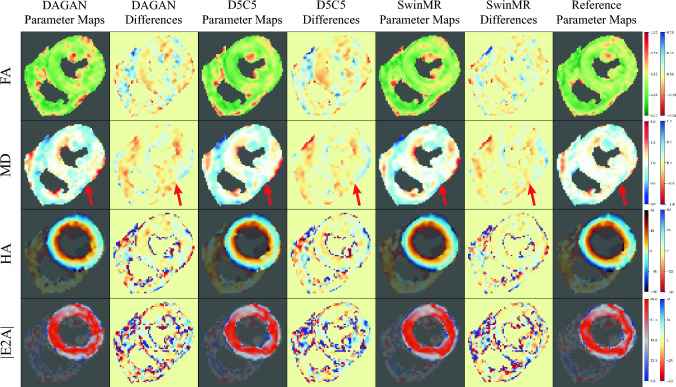
Diffusion parameter maps of the reconstruction results (AF $\times 4$) and the reference of a healthy systole case from testing set Test-S. Row 1: fractional anisotropy (FA); Row 2: mean diffusivity (MD); Row 3: helix angle (HA); Row 4: absolute value of the second eigenvector (|E2A|).

**Figure 8:**
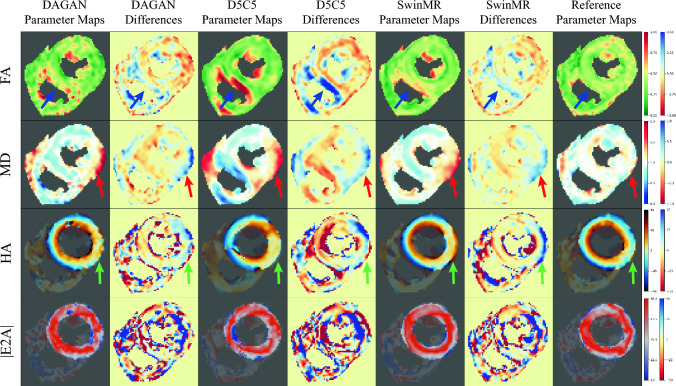
Diffusion parameter maps of the reconstruction results (AF $\times 8$) and the reference of a healthy systole case from testing set Test-S. Row 1: fractional anisotropy (FA); Row 2: mean diffusivity (MD); Row 3: helix angle (HA); Row 4: absolute value of the second eigenvector (|E2A|).

Some cases of visualised DT parameter maps are presented in this study, including FA, MD, HA and absolute value of E2A (|E2A|). The DT parameter maps of a systole healthy case from Test-S with different AFs are shown in Fig. 6 (AF $\times 2$), Fig. 7 (AF $\times 4$), and Fig. 8 (AF $\times 8$). The DT parameter maps of a diastole healthy case from Test-D with different AFs are shown in Figure S2 (AF $\times 2$), Figure S3 (AF $\times 4$), and Figure S4 (AF $\times 8$) in Supplementary. The DT parameter maps of a systole MI case from Test-MI-S with different AFs are shown in Figure S5 (AF $\times 2$), Figure S6 (AF $\times 4$), and Figure S7 (AF $\times 8$) in Supplementary. The DT parameter maps of a diastole MI case from Test-MI-D with different AFs are shown in Figure S8 (AF $\times 2$), Figure S9 (AF $\times 4$), and Figure S10 (AF $\times 8$) in Supplementary.

### Computational cost assessment

In this section, we provided the computational cost information for these deep learning-based reconstruction models. In the training stage, D5C5^15^ and DAGAN^21^ were trained on one NVIDIA RTX 3090 GPU with 24 GB GPU memory, and SwinMR^19^ was trained on two NVIDIA RTX 3090 GPUs. In the inference stage, all three models were tested on an NVIDIA RTX 3090 GPU (or an Intel Core i9-10980XE CPU). We recorded the inference time on GPU/CPU, the inference memory usage and the number of parameters (#PARAMs) to measure the computational cost. The inference time and memory usage were measured using an input of $96 \times 96 \times 1$ DWI, averaged over ten repetitions. The memory usage was the maximum usage during inference, measured by ‘nvidia-smi’. The full analysis table can be found in Supp. Table S5.

## Discussion

Cardiac DTI has demonstrated significant potential to provide insights into myocardial microstructure and impact the clinical diagnosis of cardiac diseases. However, its use is currently limited to clinical research settings, due to its low SNR and prolonged scanning time. Deep learning-based fast MRI is an emerging technique that can considerably accelerate the imaging readout. This technique directly contributes to reducing image artefacts, including EPI image distortions and cardiac motion artefacts. Additionally, it can be used to increase image spatial resolution, and potentially reduce the total scan time, as fewer repetitions may be needed.

In this study, we have investigated the performance of deep learning-based methods in the context of cDTI reconstruction. We have implemented three deep learning-based MRI reconstruction methods, namely DAGAN^21^, D5C5^15^ and SwinMR^19^, on our cDTI dataset. For the principles for the model selection, to best meet the diversity of the research subjects, we have selected one representative model from each of three different types of MRI reconstruction models. From the perspective of network structure, both CNNs and Transformers have been included. It is noted that we do not consider those multi-coil (or sensitivity maps required) models since our experiments are based on the single-channel magnitude data and retrospective *k*-space undersampling. Experimental results have been reported from the perspective of reconstruction quality assessment and DT estimation quality assessments.

According to Table 2, in the reconstruction tasks with undersampling masks of AF $\times 2$ and AF $\times 4$, D5C5 has achieved superior PSNR and SSIM, while SwinMR has achieved better deep learning-based perceptual scores, i.e., LPIPS and FID. In the reconstruction tasks at AF $\times 8$, SwinMR has outperformed other methods across all the metrics applied.

According to Fig. 5, in the reconstruction tasks of AF $\times 2$, all three methods have produced fairly good visual reconstruction results. In the reconstruction tasks of AF $\times 4$, all three methods have successfully recovered overall structure information, whereas they have behaved differently in the recovery of the high-uncertainty area. For example in the experiment on Test-S at AF $\times 4$ (Row 3–4, Col 1–5, Fig. 5), the red arrows indicate the high-uncertainty area on the LV myocardium due to the signal loss. DAGAN has provided a noisy estimation while SwinMR has clearly preserved this part of information. However, the results of D5C5 have missed the information in this area.

In reconstruction tasks of AF $\times 8$, neither of three methods has successfully produced visually satisfied reconstruction results. For example in the experiment on Test-S with AF $\times 8$ (Row 5–6, Col 1–5, Fig. 5), a large amount of visible aliasing artefacts along the PE direction has remained in the reconstruction results of both D5C5 and SwinMR, with D5C5 performing relatively worse than SwinMR. DAGAN, to some extent, has eliminated the aliasing artefacts at the expense of the increased noise, leading to a low-SNR reconstruction. Regarding the recovery of high-uncertainty area (green arrow), D5C5 has reconstructed myocardium severely affected by aliasing artefacts, but has not introduced significant hallucination. DAGAN has produced reconstruction with low SNR and tried to ‘in-paint’ the missing myocardium guided by its prior knowledge. We believe that this phenomenon is partly caused by the adversarial learning mechanism due to the tendency of GAN-based methods to introduce noise and artefacts, which has been reported in previous researches^38,53^. SwinMR has retained most information of the myocardium, whereas the reconstruction has severely affected by the hallucination. Hallucination is usually defined as the artefacts or incorrect features that occur due to the prior that cannot be produced from the measurements^54^. Based on the empirically observation, we have found such hallucination was getting stronger at high AF (difficult tasks), or when using a powerful and ‘stronger-prior’ model, for example, Transformers (SwinMR) or generative models (DAGAN).

Regarding the fidelity of the reconstruction, D5C5 has shown superiority on the condition of a relative lower AF, whereas this superiority has been observed disappearance on the condition of a relative higher AF. This phenomenon is due to the utilisation of DC module in D5C5 (Fig. 3), which combines the *k*-space measurements information with the CNN estimation to keep the consistency. According to Supp. Figure S1, in the reconstruction task at a relative lower AF, a large proportion of information in the final output of D5C5 is provided by the DC module, whereas this proportion is significantly decreased in a relative higher AF reconstruction task (AF $\times 8$). Therefore, this kind of unrolling-based methods with DC module is more suitable for the reconstruction at a relative lower AF.

For the perceptual score of the reconstructions, experiments have shown SwinMR outperforms D5C5 and DAGAN on metrics LPIPS and FID. However, even though the perceptual score has a high correlation with the observation of human, it is not always equivalent to a better reconstruction quality^38^. According to Fig. 5 (green arrow), SwinMR has learnt to estimate a ‘fake’ reconstruction detail (hallucination) for a higher perceptual score, which is totally unacceptable and dangerous for clinical use. We believe this phenomenon is caused by the nature of the Transformer applied in SwinMR, which is powerful enough to estimate and generate details that does not exist originally. In addition, the utilisation of the perceptual VGG-based loss restricts SwinMR to produce more *perceptual-similar* reconstruction instead of *pixel-wise-similar* reconstruction.

In general, the differences in tensor parameter global mean values between reference and reconstruction results tended to increase as the AF rises. Regarding the global mean values of FA, DAGAN has demonstrated superiority on the Test-S and Test-MI-S, with its superiority growing as the AF increases. On the Test-D and Test-MI-D, the three methods have yielded similar results, with no statistically significant difference observed. Regarding the global mean values of MD, D5C5 and SwinMR have outperformed DAGAN across all the testing sets. Specifically, D5C5 has delivered better results on Test-S, while SwinMR has excelled on Test-MI-S. On the Test-D and Test-MI-D, SwinMR and D5C5 have achieved similar results with no statistical difference at AF $\times 2$ and AF $\times 4$, while SwinMR has surpassed D5C5 at a higher AF (AF $\times 8$). For the global mean values of HA Slope, it is clear that SwinMR has outperformed DAGAN and D5C5 on all testing sets, with its superiority being statistically significant on Test-S and Test-D. In terms of the global mean values of E2A, generally, SwinMR has achieved better or comparable results among the three methods, but the differences are typically not statistically significant.

Generally, the quality of DT parameter maps has decreased as the AF increases. We believe that at AF $\times 2$ and AF $\times 4$, the DT parameter maps calculated by these three methods can achieve similar level with the reference. For the MI cases from out-of-the-distribution testing set Test-MI, these three methods can successfully preserve the information in lesion area for clinical use. For example at AF $\times 2$, all three methods have provided visually similar DT parameters maps with the reference (Fig. 6 and Supp. Figure S5). At AF $\times 4$, all three methods can recover most information of the DT parameters maps. DAGAN has produced produce noisier DT parameter maps, while SwinMR and D5C5 have produced the smoother DT parameter maps, which matches the results from the reconstruction quality assessment. It can be observed that, from the MD map and its corresponding error map, the vertical aliasing (along PE) direction has affected the DT parameter maps (Fig. 7, red arrows). The intensity of the MI area in the MD map of DAGAN has had a trend to decrease, while D5C5 and SwinMR has clearly preserved it (Supp. Figure S1, red arrows).

However, at AF $\times 8$, the quality of DT parameter maps has been significantly worsened, which also matches the results from the reconstruction quality assessment. For the FA map, a band of higher FA is expected to be observed in the mesocardium for a healthy heart^55^. However, DAGAN and D5C5 have failed to recover the band of higher FA, where DAGAN has produced very noisy FA map, and D5C5 over-smoothed the FA map and wrongly estimate a highlight area (Supp. Figure S7, blue arrows). For the MD map, the affect from the aliasing observed at AF $\times 4$, has become more severe. In the healthy case, the highlight area has wrongly appeared in MD maps from all three methods, which is unacceptable for clinical use and may lead to misdiagnosis (Fig. 8, red arrows). In the MI case, the MI lesion area tended to decrease for all the methods, especially for the results of DAGAN, where the lesion area has nearly disappeared (Supp. Figure S7, red arrows). For the HA map, it can be observed that SwinMR can produce relatively smooth HA map, while DAGAN can only reconstruct very noisy one. However, the direction of HA has been wrong estimated in the epicardium of the healthy case (Fig. 8, green arrows). This is not acceptable for clinical use and more likely to lead to misdiagnosis such as MI. For the |E2A| map, DAGAN tended to reconstruct a noisy map, while SwinMR tended to produce a smooth map. All three methods can produce similar results with reference even at AF $\times 8$.

Through our experiments, we have demonstrated that the models discussed in this paper can be effectively applied for clinical use at AF $\times 2$ and AF $\times 4$. However, at AF $\times 8$, the performance of these three models, including the best-performing SwinMR, has still remained limited.

We have also provided the computational cost analysis for these models (Supp. Table S5). The generator of DAGAN and SwinMR follow a non-iterative style, where DAGAN has achieved the faster inference time but a larger number of parameters, while SwinMR has longer inference time, but smaller number of parameters. This is because a large portion of operations in SwinMR are based on vector multiplication (multi-head self-attention), which are non-parametric, whereas the generator of DAGAN mostly relies on convolution and de-convolution. D5C5, as an algorithm unrolling model, is highly efficient in terms of parameters and memory usage. However, it has a longer inference time compared to DAGAN, due to its iterative style. (Although it is an end-to-end model, modules are arranged iteratively.) It is noted that D5C5 was implemented by Python library ‘Theano’, while DAGAN and SwinMR were implemented by Python library ‘PyTorch’. There might be biases in the measurement of inference time and memory usage due to different libraries.

We hope that this study will serve as a baseline for the future cDTI reconstruction model development. Our findings have indicated that there are still limitations when directly applying these general MRI reconstruction methods for cDTI reconstruction.

*There is an absence of restrictions on diffusion.* The loss functions utilised in the three models discussed in this study all rely on image domain loss, with D5C5 and DAGAN additionally incorporating the frequency domain loss and the perceptual loss. In other words, there is no diffusion information restriction implemented during the model training stage. For further studies, the diffusion tensor or parameter maps can be jointly considered into the loss function. For example, the utilisation of physics-informed loss is a potential solution for the restriction; a pre-trained neural network mapping from DWIs to DT may help to build the supervised loss for DWI reconstruction, by minimising the distance in ‘diffusion tensor’ space. Moreover, physical constraints on diffusion can be incorporated into the network. For example, the physics information like *b*-value, diffusion direction, can be fused into the network by a vector-based or a prompt-based embedding.

*There is a trade-off between perceptual performance and quantitative performance.* Cardiac diffusion tensor MRI is a quantitative technique, which places greater emphasis on contrast, pixel intensity range, and pixel-wise fidelity, also referred to as pixel-wise distance. The microstructural organisation of myocardium revealed by cDTI is sensitive to the pixel intensity of DWIs, which represents the true physiological conditions. Even minor discrepancies can lead to significant errors in interpreting the arrangement of myocardial fibres. However, the models discussed in this study were originally designed for structural MRI. Especially for DAGAN and SwinMR, they tended to pay more attention on the ‘perceptual-similarity’, which can be regarded as the latent space distance. Specifically, the perceptual loss applied for training explicitly restricts the latent space distance, while the adversarial learning mechanism implicitly minimises a statistical discrepancy between two distributions in latent space. A trade-off exists between pixel-wise fidelity and perceptual-similarity^56^. For example, blurred images generally exhibit better pixel-wise fidelity, while the images with clear but ‘fake’ details tend to have better perceptual-similarity^38^. This trade-off is observable in the visualised examples provided in Supp. Figure S11.

From another perspective, such ‘fake’ details with high perceptual score but low fidelity can be viewed as hallucinations, which are harmful for clinical use^54^. Consequently, for further studies, more efforts should be made to consider how to improve the pixel-wise fidelity rather than the perceptual-similarity, or how to prevent the appearance of the ‘fake’ information. We have found more and more researchers in the computer vision community tended to exploit larger and deeper network backbones and leverage emerging generative models for solving inverse problem including MRI reconstruction^22^. Concurrently, it is becoming essential and urgent to mitigate the incidence of hallucinations, potentially through updating the network structure, incorporating new restrictions, or adopting novel training strategies.

*There is a gap between current DT evaluation methods and the true quality of cDTI reconstruction.* This study has revealed that the global mean value of diffusion parameters is not always accurate or sensitive enough to evaluate the diffusion tensor quality. For example, Table 3 indicates no statistically significant difference in MD between reconstruction results (even including ZF) and the reference on Test-S, whereas the Fig. 8 shows that the MD maps are entirely unacceptable. This discrepancy arises because the MD value increases and decreases in different parts of the MD map, while the global mean value maintains relative consistency, rendering the global mean MD ineffective in reflecting the quality of the final DT estimation. For future work, in addition to the visualised assessment, we will applied the down-stream task assessment, e.g., utilising a pre-trained pathology classification or detection model to evaluate the reconstruction quality. Theoretically, better classification or detection accuracy corresponds to improved reconstruction results.

There are still limitations for this study. (1) The size of the testing sets is not sufficiently large. The relatively small testing sets enlarge the randomness of experimental results and reduce the reliability of statistical tests. In future studies, we will expand our dataset, especially for the patient data. According to Supp. Table S4, compared to the healthy volunteers, samples from patients in our dataset are insufficient and unbalanced. The lack of patient samples leads to the model’s inability to correctly recover the pathology information, which also results in errors in subsequent DT estimation. With sufficient patient samples, we will consider incorporating pathology information into the reconstruction model, allowing accurate DWI reconstruction and further DT estimation for different types of cardiac diseases. (2) Our simulation experiment is based on the retrospective *k*-space undersampling on single-channel magnitude DWIs that have been reconstructed by the MR scanner software. However, the raw data acquired from the scanner, prior to reconstruction, is typically multi-channel complex-value data in *k*-space. The retrospective undersampling step itself removed a large amount of high-frequency noise, leading to unrealistic post-processing results. Additionally, our experiment involves retrospective undersampling using simulation-based GRAPPA-like Cartesian *k*-space undersampling masks (Supp. Figure S1), which are inconsistent with the equal-spaced readout used in the scanner. In future studies, we aim to conduct our experiment on prospectively acquired multi-channel *k*-space raw data.

## Conclusion

In conclusion, we have investigated the application of deep learning-based methods for accelerating cDTI reconstruction, which has significant potential for improving the integration of cDTI into routine clinical practice. Our study focused on three different models, namely D5C5, DAGAN, and SwinMR, which have been evaluated on the cDTI dataset with the AF of $\times 2$, $\times 4$, and $\times 8$. The results have demonstrated that the examined models can be effectively used for clinical use at AF $\times 2$ and AF $\times 4$, with SwinMR being the recommended optimal approach. However, at AF $\times 8$, the performance of all models has remained limited, and further research is required to improve their performance at a relative higher AF.

### Supplementary Information

Supplementary Information.

## Data Availability

The data that support the findings of this study are available from Imperial College London but restrictions apply to the availability of these data, which were used under license for the current study, and so are not publicly available. Data are however available from the authors upon reasonable request and with permission of Imperial College London.
