# Supplementary material for: Deep learning-based diffusion tensor cardiac magnetic resonance reconstruction: a comparison study
Source: Sci Rep. 2024 Mar 7;14:5658. doi: 10.1038/s41598-024-55880-2 (PMC10920645; doi:10.1038/s41598-024-55880-2)
Supplement: Supplementary file 1 — Supplementary Information. [file 41598_2024_55880_MOESM1_ESM.pdf]

Supplementary of " Deep Learning-based Diffusion Tensor Cardiac Magnetic Resonance Reconstruction: A Comparison Study"

Jiahao Huang[1,2], Pedro F. Ferreira[1,2], Lichao Wang[1,3], Yinzhe Wu[1,2], Angelica I. Aviles-Rivero[4], Carola-Bibiane Schönlieb[4], Andrew D. Scott[1,2], Zohya Khalique[1,2], Maria Dwornik[1,2], Ramyah Rajakulasingam[1,2], Ranil De Silva[1,2], Dudley J. Pennell[1,2], Sonia Nielles-Vallespin[1,2,*], and Guang Yang[1,2,*]

[1]National Heart and Lung Institute, Imperial College London, London, United Kingdom
[2]Cardiovascular Research Centre, Royal Brompton Hospital, London, United Kingdom
[3]Department of Computing, Imperial College London, London, United Kingdom
[4]Department of Applied Mathematics and Theoretical Physics, University of Cambridge, Cambridge, United Kingdom
[*]Co-last senior authors. Send correspondence to {j.huang21,g.yang}@imperial.ac.uk

ABSTRACT

This is the supplementary of "Deep Learning-based Diffusion Tensor Cardiac Magnetic Resonance Reconstruction: A Comparison Study".

Methodology

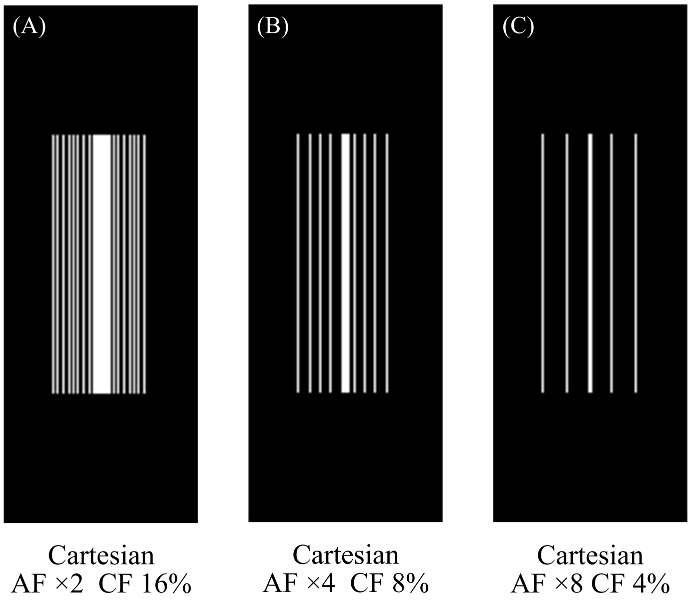

Figure S1. Three Cartesian *k*-space undersampling masks applied in this work. AF: acceleration factor; CF: centre factor.

Table S1. The implementation detail of DAGAN. '#': 'the number of'; Conv: convolutional layer.

Implementation Detail of DAGAN	
**Network Parameter (Generator)**	
Input Shape	$96 \times 96 \times 1$
Output Shape	$96 \times 96 \times 1$
#Conv Layer	16
Conv Kernel Size	3×3
Initial Embedding Channel	64
Max Embedding Channel	1024
**Network Parameter (Discriminator)**	
Input Shape	$96 \times 96 \times 1$
Output Shape	1
#Conv Layer	11
Conv Kernel Size	3×3
Initial Embedding Channel	64
Max Embedding Channel	1024
#Feature (for Classification Head)	4608
**Optimisation Parameter**	
Max Epoch	1000
Batchsize	24
Loss #1 (Type)	Image space L2 Loss
Loss #1 (Weight)	15
Loss #2 (Type)	Frequency space L2 Loss
Loss #2 (Weight)	0.1
Loss #3 (Type)	Perceptual (VGG) L2 Loss
Loss #3 (Weight)	0.0025
Loss #4 (Type)	Adversarial BCE Loss
Loss #4 (Weight)	1
Optimiser	Adam
Initial Learning Rate	1e-4
Learning Rate Schedule	StepLR
Learning Rate Decay Interval (Epoch)	10
Learning Rate Decay Rate	0.8
Early Stopping Number (Epoch)	8

Table S2. The implementation detail of D5C5. '#': 'the number of'; Conv: convolutional layer.

Implementation Detail of D5C5	
**Network Parameter**	
Input Shape	$256 \times 96 \times 1$
Output Shape	$256 \times 96 \times 1$
#Stage/Cascade	5
#Conv (in Each Stage/Cascade)	5
**Optimisation Parameter**	
Batchsize	16
Loss (Type)	Image space L2 Loss
Optimiser	Adam
Initial Learning Rate	1e-3
Learning Rate Schedule	StepLR

Table S3. The implementation detail of SwinMR. '#': 'the number of'; RSTB: residual Swin Transformer block; STL: Swin Transformer layer; Head: the attention head in multi-head self-attention; Window Size: the attention head in window-based multi-head self-attention.

Implementation Detail of SwinMR	
**Network Parameter**	
Input Shape	$96 \times 96 \times 1$
Output Shape	$96 \times 96 \times 1$
#RSTB	6
#STL	6
#Head	6
Window Size	8
Embedding Channel	180
**Optimisation Parameter**	
Total Training Step	100000
Batchsize	8
Loss #1 (Type)	Image space Charbonnier Loss (eps: 1e-9)
Loss #1 (Weight)	15
Loss #2 (Type)	Frequency space Charbonnier Loss (eps: 1e-9)
Loss #2 (Weight)	0.1
Loss #3 (Type)	Perceptual (VGG) L1 Loss
Loss #3 (Weight)	0.0025
Optimiser	Adam
Initial Learning Rate	2e-4
Learning Rate Schedule	MultiStepLR
Learning Rate Milestone (Step)	50000, 70000, 90000
Learning Rate Decay Rate	0.5

Table S4. The detailed information of the dataset. AMYLOID: amyloidosis; DCM: dilated cardiomyopathy; rDCM: in-recovery DCM; HCM: hypertrophic cardiomyopathy HCM G+P-: HCM genotype-positive–phenotype-negative MI: acute myocardial infarction.

Disease Type	Cardiac Phase	Total	Train & Validation						Test
			All Folds	Fold-1	Fold-2	Fold-3	Fold-4	Fold-5	
Health	Diastole	112	90	18	18	18	18	18	22
Health	Systole	129	103	21	21	21	20	20	26
AMYLOID	Diastole	14	11	3	2	2	2	2	3
AMYLOID	Systole	17	14	3	3	3	3	2	3
DCM	Diastole	23	18	4	4	4	3	3	5
DCM	Systole	24	19	4	4	4	4	3	5
HCM (G+P-)	Diastole	24	19	4	4	4	4	3	5
HCM (G+P-)	Systole	24	19	4	4	4	4	3	5
HCM	Diastole	23	18	4	4	4	3	3	5
HCM	Systole	16	13	3	3	3	2	2	3
rDCM	Diastole	16	13	3	3	3	2	2	3
rDCM	Systole	19	15	3	3	3	3	3	4
MI	Diastole	20	0	0	0	0	0	0	20
MI	Systole	20	0	0	0	0	0	0	20

Experiments and Results

Table S5. The quantitative assessment of computational cost, including the inference time on GPU and CPU, the inference memory usage on GPU, and the number of parameters (#PARAMs) The model inferences were conducted on one NVIDIA RTX 3090 GPU or (or an Intel Core i9-10980XE CPU). The inference time and memory usage were measured using an input of $96 \times 96 \times 1$ DWI, averaged over ten repetitions. The memory usage was the maximum usage during inference, which measured by 'nvidia-smi'. For DAGAN, only the generator was used in the inference stage, and its #PARAMs are quoted as 'generator (generator and discriminator)'. It is noted that D5C5 was implemented by Python library 'Theano' while D5GAN and SwinMR were implemented by Python library 'PyTorch'. There might be biases in the measurement of inference time and memory usage during to the optimisation of different library.

**Methods**	Inference Time (s)		Inference Memory Usage (MiB)	#PARAMs (M)
	CPU	GPU		
D5C5	0.331 (0.000)	0.011 (0.000)	583	0.566
DAGAN	0.030 (0.002)	0.002 (0.000)	1979	60.052 (88.642)*
SwinMR	0.621 (0.008)	0.042 (0.000)	1936	11.402

Table S6. Differences of diffusion tensor parameter global mean values between the reference and reconstruction results (undersampled k-space zero-filled images ZF included), on diastole testing sets Test-D and Test-MI-D. Mean absolute error are applied for fractional anisotropy (FA), mean diffusivity (MD), and mean absolute angular error are applied for helix angle gradient (HA Slope) and second eigenvector (E2A). The results are quoted as 'median [interquartile range]'. * indicates the specific error distribution is significantly different from the **best-resulting**distribution by Mann-Whitney Test ($p < 0.05$). Data point with a green background indicates that the specific distribution of corresponding diffusion tensor parameter global mean values is NOT significantly different from the reference distribution by Mann-Whitney Test ($p > 0.05$). Units: FA unitless; MD $10^{-3} \cdot \text{mm}^2 \cdot \text{sec}^{-1}$; HA Slope degrees $\cdot$ mm^{-1} and E2A degrees.

**DT Para.**	**Test-D**				**Test-MI-D**			
**AF** ×2	ZF	DAGAN	D5C5	SwinMR	ZF	DAGAN	D5C5	SwinMR
FA	0.044 [0.017] ⋆	0.007 [0.011]	0.005 [0.006]	**0.004 [0.004]**	0.058 [0.019] ⋆	**0.007 [0.011]**	0.010 [0.009]	0.008 [0.007]
MD	0.018 [0.023] ⋆	0.013 [0.013]	**0.007 [0.010]**	0.009 [0.009]	0.042 [0.017] ⋆	0.007 [0.012]	0.007 [0.006]	**0.004 [0.006]**
HA Slope	2.188 [1.105] ⋆	0.370 [0.522] ⋆	0.238 [0.295]	**0.218 [0.377]**	1.961 [1.065] ⋆	0.315 [0.290]	0.173 [0.256]	**0.167 [0.274]**
E2A	3.124 [3.340] ⋆	0.887 [1.259] ⋆	0.524 [0.796]	**0.497 [0.661]**	3.609 [3.316] ⋆	0.693 [1.246]	**0.608 [0.781]**	0.895 [0.821]
**AF** ×4	ZF	DAGAN	D5C5	SwinMR	ZF	DAGAN	D5C5	SwinMR
FA	0.120 [0.041] ⋆	0.013 [0.017]	0.013 [0.022]	**0.009 [0.012]**	0.138 [0.024] ⋆	0.011 [0.009]	0.016 [0.018]	**0.007 [0.014]**
MD	0.075 [0.057] ⋆	0.029 [0.030] ⋆	**0.014 [0.022]**	0.015 [0.014]	0.130 [0.032] ⋆	0.036 [0.031] ⋆	**0.014 [0.018]**	0.015 [0.017]
HA Slope	2.783 [1.035] ⋆	0.641 [0.977] ⋆	0.592 [0.896] ⋆	**0.392 [0.643]**	3.076 [0.699] ⋆	0.508 [0.810]	0.682 [0.294] ⋆	**0.215 [0.483]**
E2A	5.093 [3.850] ⋆	1.464 [1.922]	1.422 [1.881]	**1.036 [1.200]**	5.006 [5.062] ⋆	1.407 [1.566]	1.296 [1.195]	**1.061 [1.690]**
**AF** ×8	ZF	DAGAN	D5C5	SwinMR	ZF	DAGAN	D5C5	SwinMR
FA	0.233 [0.080] ⋆	0.035 [0.044]	**0.030 [0.034]**	0.033 [0.042]	0.248 [0.028] ⋆	**0.026 [0.043]**	0.056 [0.061]	0.034 [0.038]
MD	0.103 [0.094] ⋆	0.085 [0.099] ⋆	0.068 [0.068] ⋆	**0.041 [0.066]**	0.232 [0.065] ⋆	0.145 [0.067] ⋆	0.086 [0.067] ⋆	**0.043 [0.069]**
HA Slope	3.282 [1.301] ⋆	3.014 [2.634] ⋆	2.145 [1.883] ⋆	**1.116 [1.408]**	3.802 [1.226] ⋆	3.101 [1.453] ⋆	2.708 [1.591] ⋆	**1.599 [1.909]**
E2A	3.538 [4.984] ⋆	4.100 [4.677]	8.363 [10.243] ⋆	**3.118 [6.069]**	4.025 [3.634] ⋆	**2.476 [4.193]**	5.369 [6.679] ⋆	3.854 [5.843]

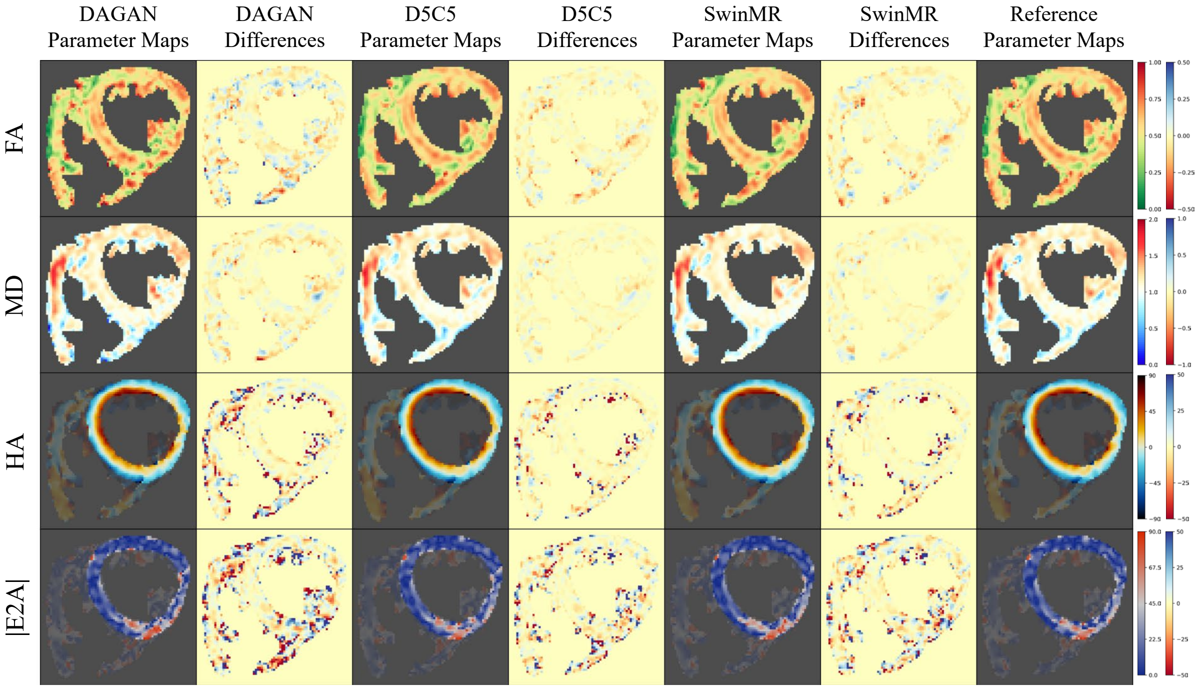

Figure S2. Diffusion parameter maps of the reconstruction results (AF ×2) and the reference of a healthy diastole case from testing set Test-D. Row 1: fractional anisotropy (FA); Row 2: mean diffusivity (MD); Row 3: helix angle (HA); Row 4: absolute value of the second eigenvector (|E2A|).

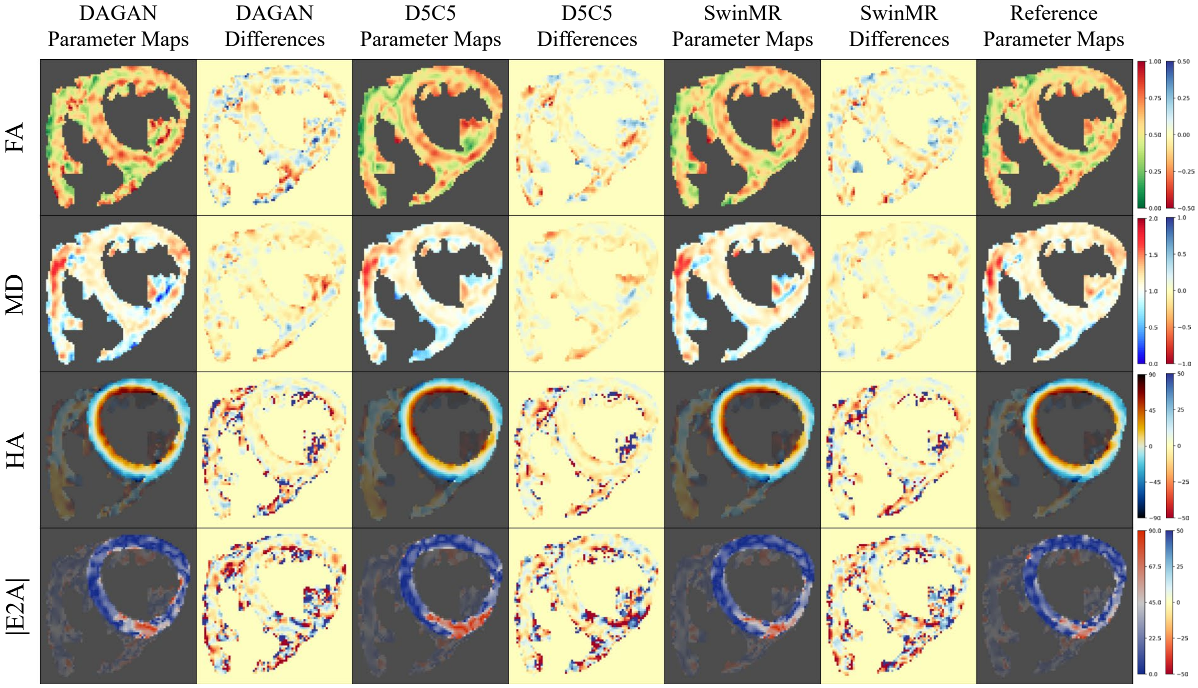

Figure S3. Diffusion parameter maps of the reconstruction results (AF ×4) and the reference of a healthy diastole case from testing set Test-D. Row 1: fractional anisotropy (FA); Row 2: mean diffusivity (MD); Row 3: helix angle (HA); Row 4: absolute value of the second eigenvector (|E2A|).

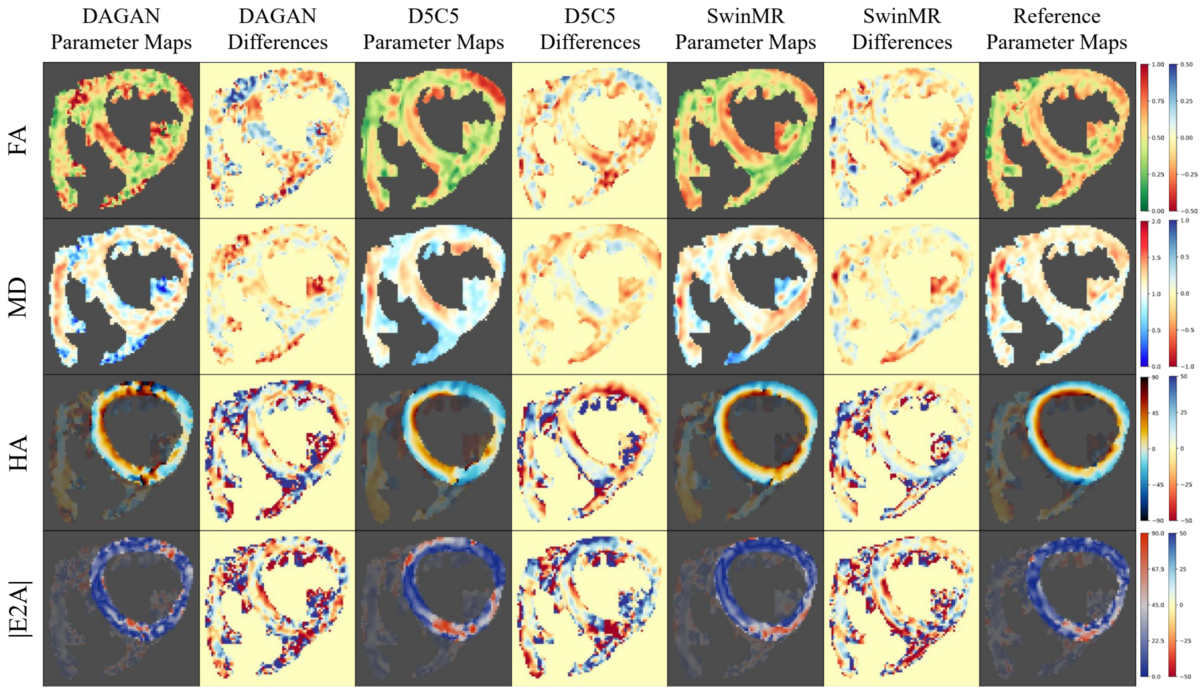

Figure S4. Diffusion parameter maps of the reconstruction results (AF $\times 8$) and the reference of a healthy diastole case from testing set Test-D. Row 1: fractional anisotropy (FA); Row 2: mean diffusivity (MD); Row 3: helix angle (HA); Row 4: absolute value of the second eigenvector (|E2A|).

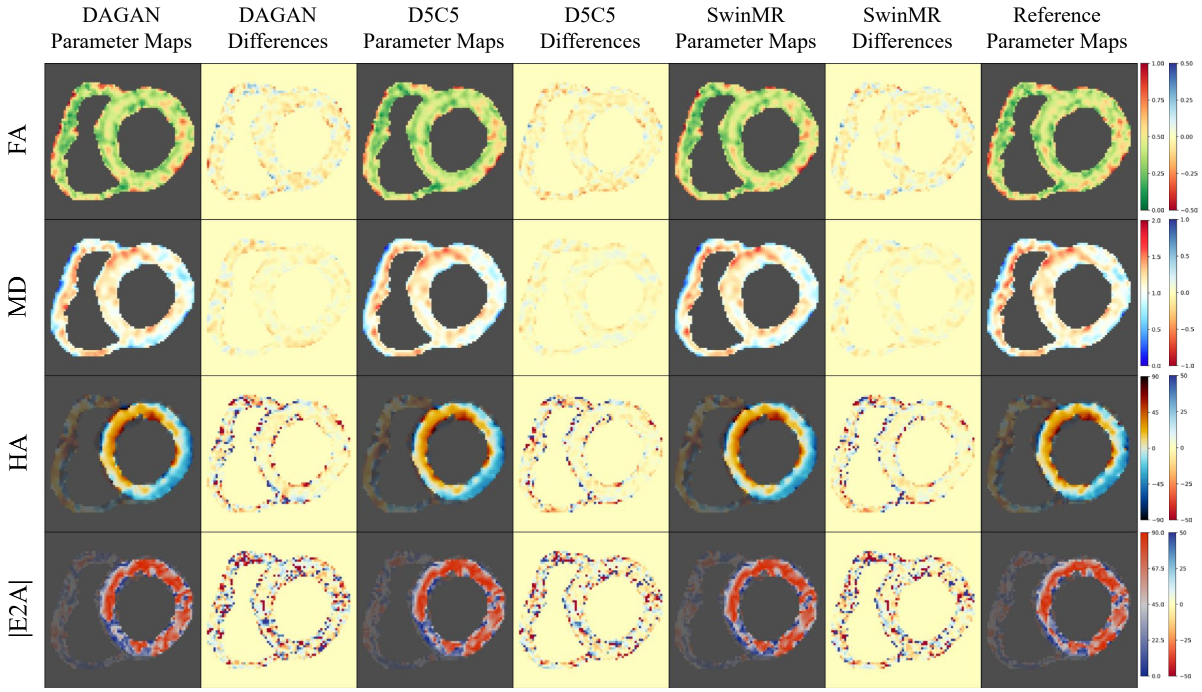

Figure S5. Diffusion parameter maps of the reconstruction results (AF $\times 2$) and the reference of a acute myocardial infarction (MI) systole case from testing set Test-MI-S. Row 1: fractional anisotropy (FA); Row 2: mean diffusivity (MD); Row 3: helix angle (HA); Row 4: absolute value of the second eigenvector (|E2A|).

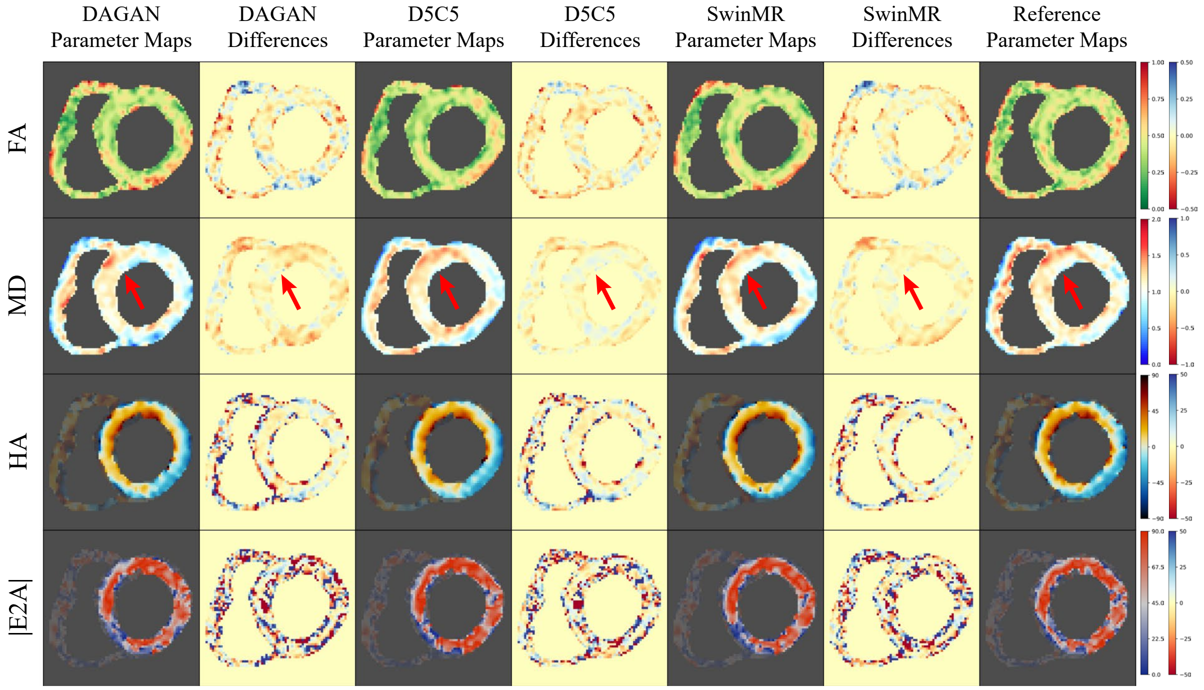

Figure S6. Diffusion parameter maps of the reconstruction results (AF ×4) and the reference of a acute myocardial infarction (MI) systole case from testing set Test-MI-S. Row 1: fractional anisotropy (FA); Row 2: mean diffusivity (MD); Row 3: helix angle (HA); Row 4: absolute value of the second eigenvector (|E2A|).

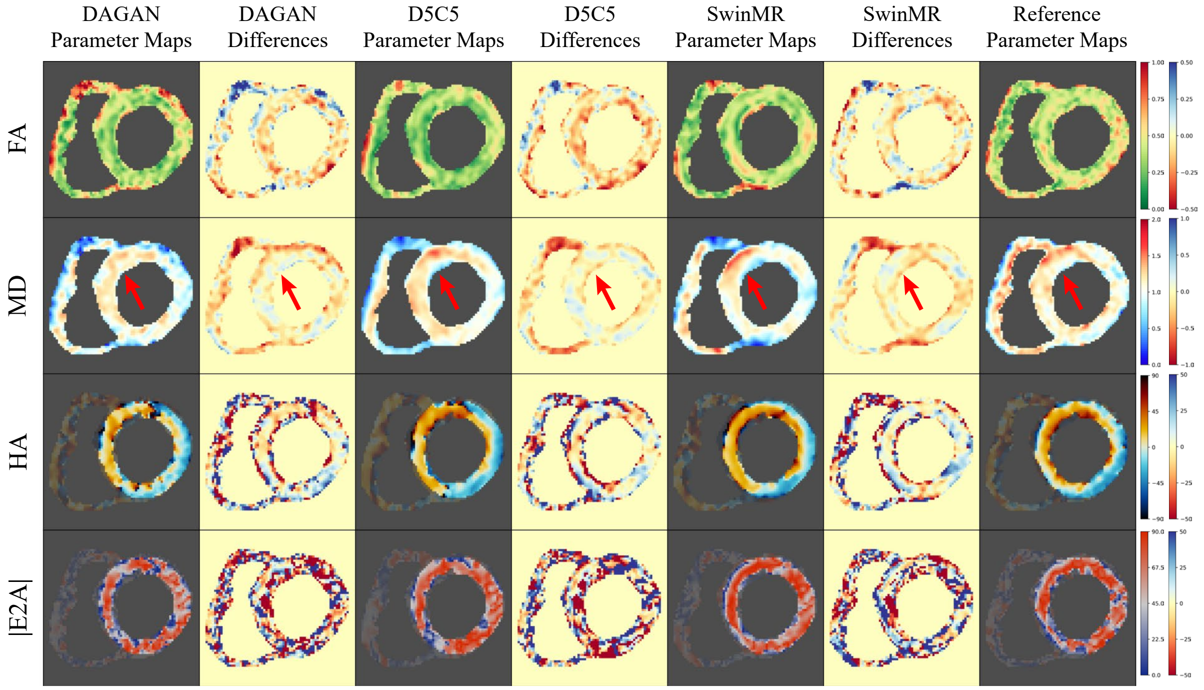

Figure S7. Diffusion parameter maps of the reconstruction results (AF ×8) and the reference of a acute myocardial infarction (MI) systole case from testing set Test-MI-S. Row 1: fractional anisotropy (FA); Row 2: mean diffusivity (MD); Row 3: helix angle (HA); Row 4: absolute value of the second eigenvector (|E2A|).

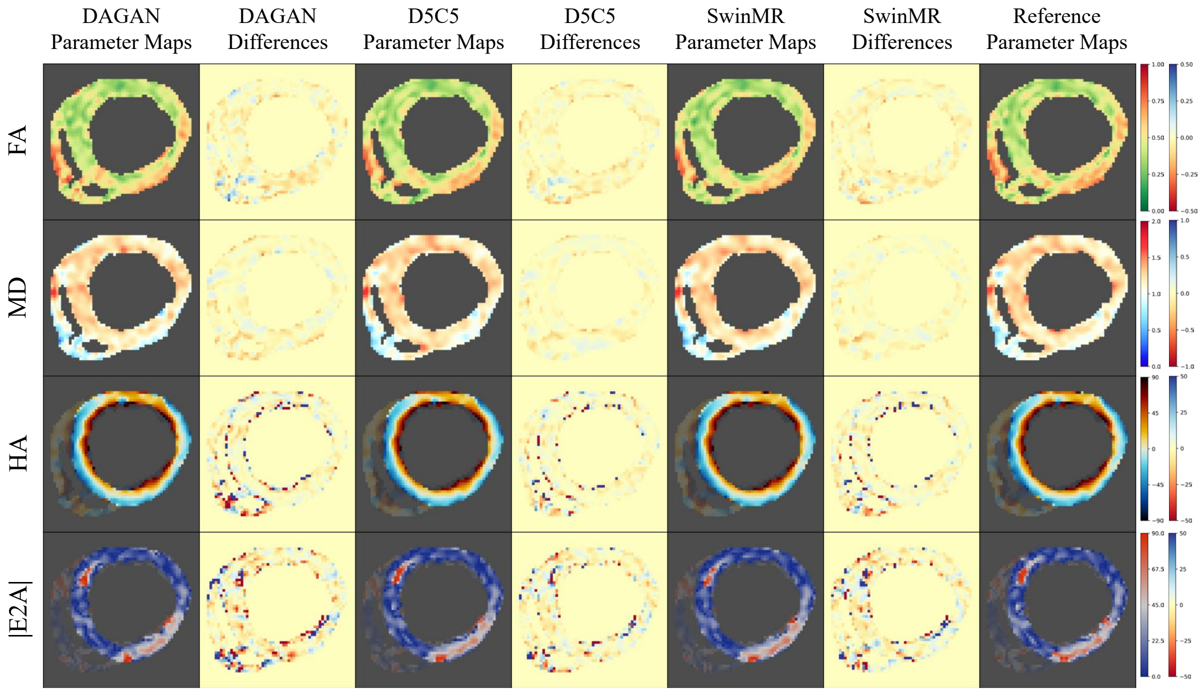

Figure S8. Diffusion parameter maps of the reconstruction results (AF $\times 2$) and the reference of a acute myocardial infarction (MI) diastole case from testing set Test-MI-D. Row 1: fractional anisotropy (FA); Row 2: mean diffusivity (MD); Row 3: helix angle (HA); Row 4: absolute value of the second eigenvector (|E2A|).

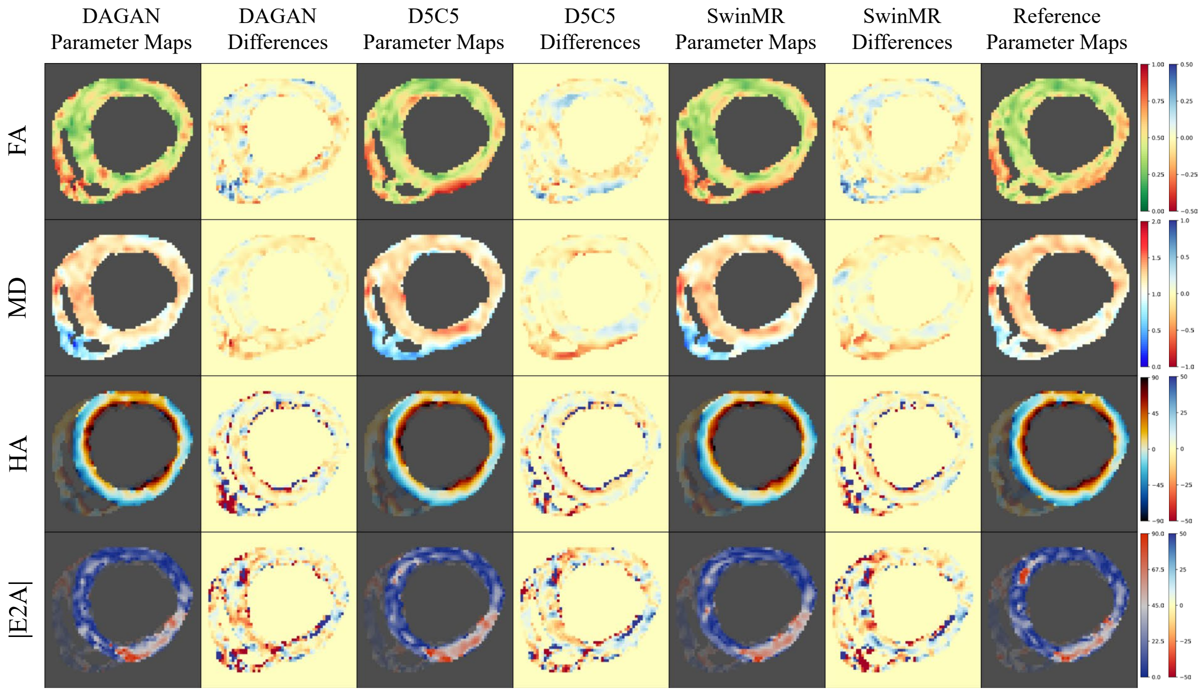

Figure S9. Diffusion parameter maps of the reconstruction results (AF $\times 4$) and the reference of a acute myocardial infarction (MI) diastole case from testing set Test-MI-D. Row 1: fractional anisotropy (FA); Row 2: mean diffusivity (MD); Row 3: helix angle (HA); Row 4: absolute value of the second eigenvector (|E2A|).

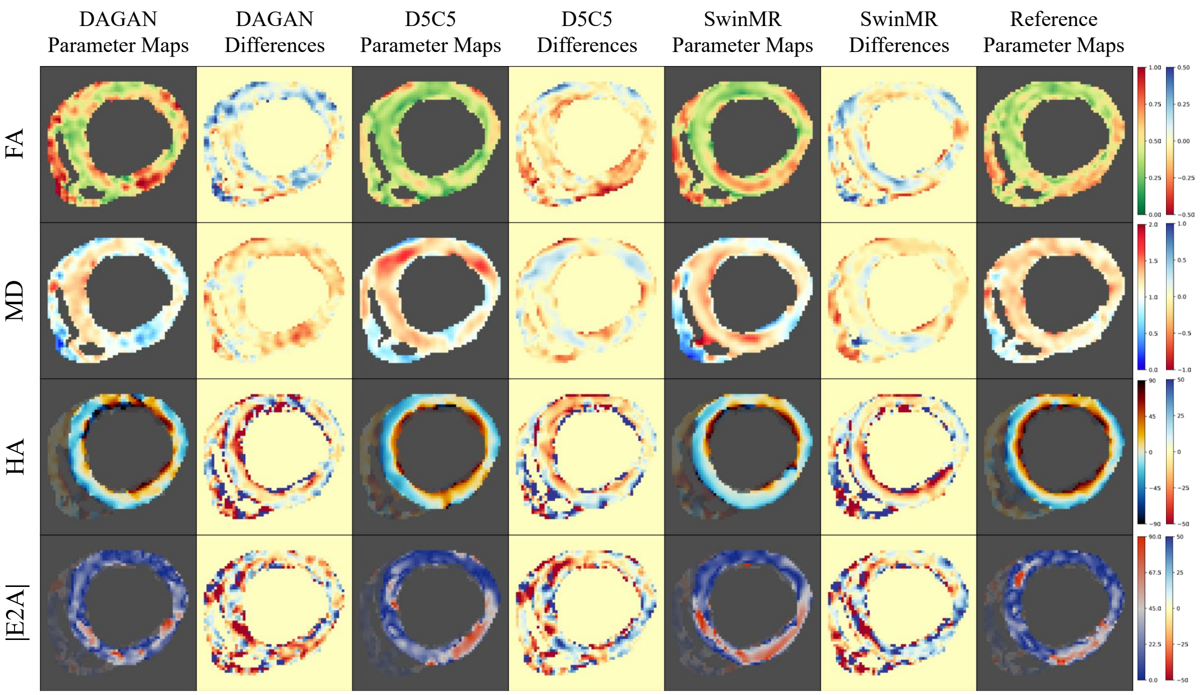

Figure S10. Diffusion parameter maps of the reconstruction results (AF ×8) and the reference of a acute myocardial infarction (MI) diastole case from testing set Test-MI-D. Row 1: fractional anisotropy (FA); Row 2: mean diffusivity (MD); Row 3: helix angle (HA); Row 4: absolute value of the second eigenvector (|E2A|).

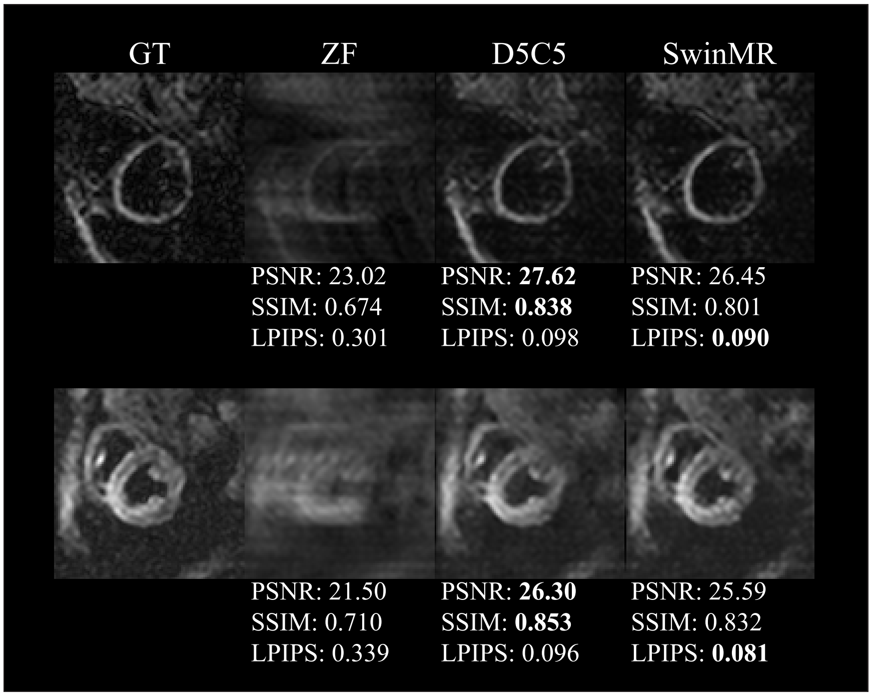

Figure S11. Visualised examples for the perception-distortion trade-off. From left to right are the ground truth (GT), undersampled k-space zero-filled images (ZF) with an acceleration factor of ×4, and the reconstruction results of D5C5 and SwinMR. Corresponding PSNR, SSIM and LPIPS are provided.
